# Identification of *Plasmodium vivax* Proteins with Potential Role in Invasion Using Sequence Redundancy Reduction and Profile Hidden Markov Models

**DOI:** 10.1371/journal.pone.0025189

**Published:** 2011-10-03

**Authors:** Daniel Restrepo-Montoya, David Becerra, Juan G. Carvajal-Patiño, Alvaro Mongui, Luis F. Niño, Manuel E. Patarroyo, Manuel A. Patarroyo

**Affiliations:** 1 Bioinformatics and Intelligent Systems Research Laboratory - BIOLISI, Universidad Nacional de Colombia, Bogotá D.C., Colombia; 2 Research Group on Combinatorial Algorithms - ALGOS-UN, Universidad Nacional de Colombia, Bogotá D.C., Colombia; 3 School of Medicine and Health Sciences, Universidad del Rosario, Bogotá D.C., Colombia; 4 Fundación Instituto de Inmunología de Colombia - FIDIC, Bogotá D.C., Colombia; 5 School of Medicine, Universidad Nacional de Colombia, Bogotá D.C., Colombia; National Institutes of Health, United States of America

## Abstract

**Background:**

This study describes a bioinformatics approach designed to identify *Plasmodium vivax* proteins potentially involved in reticulocyte invasion. Specifically, different protein training sets were built and tuned based on different biological parameters, such as experimental evidence of secretion and/or involvement in invasion-related processes. A profile-based sequence method supported by hidden Markov models (HMMs) was then used to build classifiers to search for biologically-related proteins. The transcriptional profile of the *P. vivax* intra-erythrocyte developmental cycle was then screened using these classifiers.

**Results:**

A bioinformatics methodology for identifying potentially secreted *P. vivax* proteins was designed using sequence redundancy reduction and probabilistic profiles. This methodology led to identifying a set of 45 proteins that are potentially secreted during the *P. vivax* intra-erythrocyte development cycle and could be involved in cell invasion. Thirteen of the 45 proteins have already been described as vaccine candidates; there is experimental evidence of protein expression for 7 of the 32 remaining ones, while no previous studies of expression, function or immunology have been carried out for the additional 25.

**Conclusions:**

The results support the idea that probabilistic techniques like profile HMMs improve similarity searches. Also, different adjustments such as sequence redundancy reduction using Pisces or Cd-Hit allowed data clustering based on rational reproducible measurements. This kind of approach for selecting proteins with specific functions is highly important for supporting large-scale analyses that could aid in the identification of genes encoding potential new target antigens for vaccine development and drug design. The present study has led to targeting 32 proteins for further testing regarding their ability to induce protective immune responses against *P. vivax* malaria.

## Introduction

Human malaria is caused by five parasite species from the genus *Plasmodium*, of which *Plasmodium falciparum* has a preferential distribution in African countries and is particularly important, since it produces most of the fatal cases. The second species in clinical importance for humans is *Plasmodium vivax* (predominantly distributed throughout Asia and America). *P. vivax* does not cause such an imminent life-threatening condition as that caused by *P. falciparum*; however, it imposes an important social and economic toll on the world's poorest countries, as reflected in the large number of disability adjusted life years (DALYs) associated with its incidence [1]. Furthermore, several aspects still hamper the total eradication of this disease, which include (1) the gradual emergence of antimalarial drug resistance among parasite strains, as well as (2) insecticide-resistant populations of the malaria mosquito vector, and (3) the lack of an effective vaccine [2].

Progress in *P. vivax* research has been notably delayed by contrast with *P. falciparum*, partly due to the difficulty of establishing a long-term *in vitro* culture of this species given that it is restricted to invading human reticulocytes which only account for ∼1–2% of circulating red blood cells. This difficulty has been reflected in the delayed release of its genome sequence [3], the transcriptional profile of its intra-erythrocyte developmental cycle [4] and the partial proteome of its schizont stage [5] compared to the release of the same studies in *P. falciparum*. *In silico* approaches have emerged from such experimental limitations, thereby promoting the development of an automatic analysis of the *P. vivax* transcriptome aimed at identifying parasite genes that have dominant transcription during schizont stages, mainly 30–48 hours post-infection, based on the rationale that they could be encoding proteins involved in *P. vivax* invasion of human reticulocytes [4].

Hidden Markov models (HMMs) are among the most effective and efficient approaches for analyzing large sets of biological data [6]–[8]. These models have been applied over the last 20 years in sequence analysis, gene finding and protein family characterization [9]. Chen *et al.*, designed a predictor for secreted proteins using HMM-based methods in 2003 to identify signal peptides and transmembrane proteins [10]; in 2009 Tinhosolo *et al.*, identified carotenoid genes in *P. falciparum* by comparing biosynthesis-related genes against a local database of *P. falciparum* genes using BLAST and HMM [11]. In the same year, Gaskell *et al.*., identified secreted enzymes in *Toxoplasma gondii* using SHARKhunt which used profile HMM based on the PRIAM polypeptide profile library to search for a set of genomic DNA sequences potentially encoding such proteins [12]. In 2010, Arena *et al.*, identified 16 new genes encoding trypsin proteases in 8 Apicomplexan genomes [13]. Ghouila *et al.*, constructed a system for identifying protein domains based on comparing HMM and Pfam profiles so that a Pfam family was represented by a multiple sequence alignment and a profile HMM [14].

More specifically, different HMM-based approaches have been proposed for the search, identification and characterization of new vaccine candidate proteins in the Plasmodium genus. A method for detecting new protein domains in *P. falciparum* using co-occurrence was proposed by Terrapon *et al.*, in 2009 [15]. This method has been claimed to improve the sensitivity of Pfam domain detection. More recently, Bischoff and Vaquero, in 2010 [16], reported a set of proteins potentially involved in the transcriptional machinery of the Plasmodium genus, based on *in silico* reports and databases. Particularly, a directory of factors associated with Plasmodium transcription was built by scanning the Plasmodium genome using profile HMMs. It should be stressed that HMMER software was used for the searches related to both methods [17].

One of the most used profile HMM-based tools is HMMER v3.0 which is a new generation of sequence homology search software that is used for querying sequence databases for protein homologues and performing protein alignment. Compared to BLAST, FASTA and other sequence alignment and database search tools based on classical scoring methodology, HMMER has been shown to be significantly more accurate and better at detecting remote homologues because of the strength of its underlying mathematical models. In the past, this strength came at significant computational expense, but in the new HMMER3 project, HMMER is now essentially as fast as BLAST [18].

Several adjustments to the input data are required as part of the process for obtaining the different probabilistic profile HMMs taking into account that, if there is a high degree of similarity among the biological sequences, any analysis could be biased due to redundancy. Different efficient algorithms allow a set of sequences to be constructed with a degree of similarity below a given parameter. Cd-hit uses an algorithm based on short word filtering (i.e. dipeptides, tripeptides, etc.) to determine protein similarity taking into account the minimum number of identical short substrings [19]. On the other hand, PISCES [20] uses PSI-BLAST and an inclusion/exclusion label process that is based on the size of the parameter that determines the similarity threshold.

This study describes an approach to the large-scale identification and selection of proteins playing an active role in *P. vivax* invasion of human reticulocytes. It is based on identifying secreted and/or invasion-related proteins using sequence redundancy reduction and profile HMMs to analyze genome-annotated genes with significantly high levels of transcription toward the end of the intra-erythrocyte cycle. The preference for this transcriptional stage was based on the premise that gene expression is timely regulated and therefore biological functions can be assigned to a particular instant of the parasite's developmental cycle inside the infected red blood cell [4]. The selected candidates were further analyzed; this included reviewing published experimental evidence of role during invasion and/or immunological assays leading to their classification as vaccine candidates and experimental confirmation of protein presence. Supporting information based on the prediction of subcellular localization and signal sequence, number of transmembrane regions, identification of glycosylphosphatidylinositol (GPI)–anchor sites and functional domains related to invasion, and prediction of adhesion activity was also assessed. The objective was to define robust selection criteria based on a bioinformatics strategy to find *P. vivax* proteins likely to be interesting vaccine candidates.

## Results

**Figure 1:**
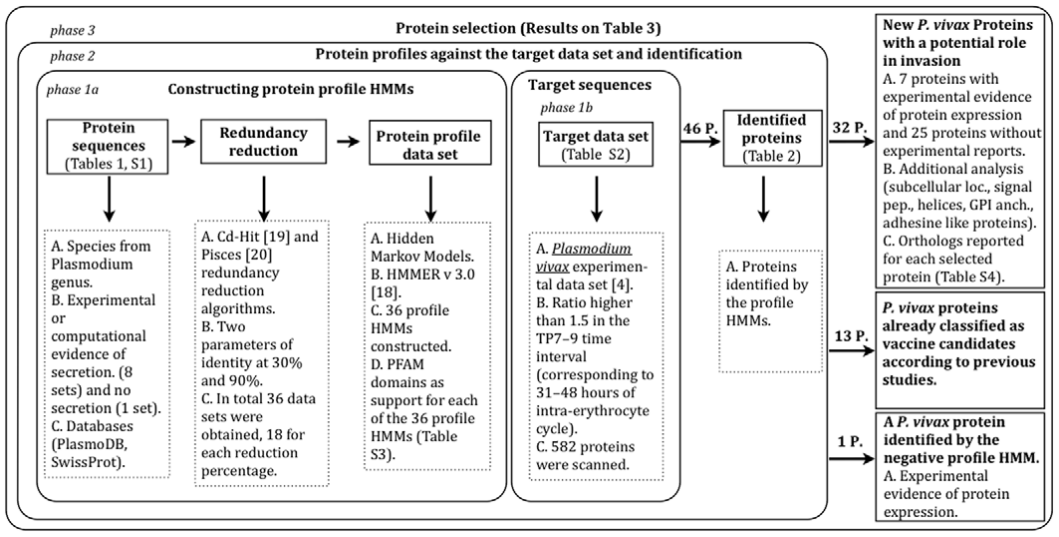
Methodology for identifying *Plasmodium vivax* proteins having a potential role in invasion using sequence redundancy reduction and profile hidden Markov models.

The results were based on the 3 phases proposed in the methodology (Figure 1). The first one consisted of constructing 36 profile HMMs and a target data set of 582 *P. vivax* open reading frames (ORFs) with predominant transcription toward the end of the intra-erythrocyte cycle. In the second phase (identification), the 36 profile HMMs were used for exploring the target data set, leading to the discovery of 46 *P. vivax* ORFs scanned by the profile HMMs. Forty-five of them encoded potentially secreted and/or invasion-related proteins, while the remaining one belonged to the negative set. It is worth noting that ORFs were included irrespective of whether they were identified once or several times. The resulting ORFs/proteins were screened in the third phase according to a literature review of published evidence regarding their secretion or previous use as vaccine candidates.

### Phase 1: Constructing protein profile HMMs (1a) and target data set (1b)

Nine protein sequence data sets were also constructed to create the protein profile HMMs. It should be highlighted that data sets had different construction criteria, were processed by 2 different redundancy reduction algorithms and that cut-off values were established at 2 different identity percentages, leading to 36 profiles in total being obtained (Table 1).

**Table 1:** Plasmodium protein data sets used for profile HMMs construction.

			Protein existence**				Organism			
# of proteins	Type	Query	Prot/Trans	Predicted	Putative	Homology	*P. falciparum*	*P. vivax*	*P. berghei*	*P. yoelii*
129*	Neg	Database	34	1	0	94	75	10	12	12
133	Pos	Database	21	81	22	9	79	28	15	11
118	Pos	Lit/DB	20	69	22	7	66	28	14	10
110	Pos	Database	21	81	0	1	71	21	8	10
88	Pos	Lit/DB	20	69	0	0	54	20	6	8
68	Pos	Lit/DB	0	68	0	0	48	11	4	5
67	Pos	Literature	6	48	8	5	67	0	0	0
42	Pos	Lit/DB	20	0	22	0	12	16	10	4
20	Pos	Lit/DB	20	0	0	0	6	9	2	3

*The remaining protein sequences correspond to *P. chabaudi* (10), *P. knowlesi* (8), *P. gallinaceum* (1) and *P. malariae* (1).

**Level of evidence that supports the existence of the protein concerned. “Prot/Trans” indicates that there is clear experimental evidence for the existence of the protein or that expression data indicate the existence of a transcript. “Homology” indicates that the existence of a protein is probable because orthologs exist in closely-related species. “Predicted” is used for entries without evidence at protein, transcript, or homology levels. ‘Putative’ indicates putative proteins having predicted existence.

Table 2 shows the ORFs/proteins identified by the 36 profile HMMs that were constructed from 9 Plasmodium protein data sets (Table S1); 8 of them were considered as secreted and some have been reported as being involved in invasion, while the remaining set contained the non-secreted proteins.

**Table 2:** Computational protein identification results.

	Protein profile datasets																																			
	20				42				67				68				88				110				118				133				129			
	cd-hit		pisces		cd-hit		pisces		cd-hit		pisces		cd-hit		pisces		cd-hit		pisces		cd-hit		pisces		cd-hit		pisces		cd-hit		pisces		cd-hit		pisces	
ID proteins	a	b	a	b	a	b	a	b	a	b	a	b	a	b	a	b	a	b	a	b	a	b	a	b	a	b	a	b	a	b	a	b	a	b	a	b
PVX_090110																																	x	x	x	x
PVX_117880					x	x	x	x	x	x	x	x	x	x	x	x	x	x	x	x	x	x	x	x	x		x	x	x	x	x	x				
PVX_003905																					x	x	x	x					x		x	x				
PVX_092975					x	x	x	x			x		x	x			x			x	x	x	x	x			x		x	x	x	x				
PVX_121920					x	x	x	x	x	x	x		x		x	x									x		x		x	x						
PVX_003800	x	x	x	x	x	x	x	x		x												x		x						x		x				
PVX_003825	x	x	x	x		x	x	x		x												x		x						x						
PVX_121885										x				x		x		x				x		x		x				x						
PVX_003805	x	x	x	x		x	x	x		x												x		x						x	x					
PVX_003850	x	x	x	x		x	x	x		x												x		x						x	x	x				
PVX_002510									x	x	x	x	x	x	x	x	x	x	x	x					x	x	x	x		x						
PVX_099980													x			x		x		x				x		x		x		x		x				
PVX_097670													x		x		x		x	x			x			x					x					
PVX_097720									x				x	x	x		x		x	x			x			x	x	x			x					
PVX_097680																												x			x					
PVX_098585					x	x	x	x																	x											
PVX_097715									x	x	x	x	x	x		x	x	x		x					x	x	x	x								
PVX_090210													x												x											
PVX_096990												x	x		x				x						x	x	x									
PVX_097705									x			x		x	x	x			x						x	x	x	x								
PVX_084720									x								x								x											
PVX_117230																			x							x										
PVX_123105									x																	x										
PVX_097700												x				x											x									
PVX_097695											x			x	x	x	x										x	x								
PVX_097710																											x									
PVX_097675										x		x		x				x									x									
PVX_101555																		x										x								
PVX_003830	x	x	x	x		x	x	x		x										x		x		x												
PVX_085930	x	x	x	x	x	x	x	x																x												
PVX_112665																	x																			
PVX_101605									x						x		x			x																
PVX_092995																	x																			
PVX_123550																	x																			
PVX_118525																		x																		
PVX_081810																			x																	
PVX_101505																				x																
PVX_109280																				x																
PVX_094425														x																						
PVX_092425									x						x																					
PVX_086850									x																											
PVX_081845									x																											
PVX_082695					x		x																													
PVX_003815		x		x		x		x																												
PVX_097590		x		x			x																													
PVX_080305	x																																			

The characters “a” and “b” represent 30% and 90% identity thresholds, respectively.

The target data set was constructed from proteins encoded in the *P. vivax* genome showing a peak transcription at the end of the intra-erythrocyte development cycle and which could therefore have potential roles in the invasion of reticulocytes [4]. Consequently, this data set only included genes exhibiting a Cy5/Cy3 expression ratio larger than 1.5 at the end of the intra-erythrocyte cycle (31–48 hours post-invasion) (Table S2).

### Phase 2: Protein profiles against the target data set and identification step

The analysis shows that, even though some sequences were shared between profile HMMs, the different modifications applied to the 9 protein data sets led to different results being obtained in all cases, except for the negative profile, in which all the 4 profile HMM modifications pointed to the same protein (PVX_090110), as will be further explained below (Table 2).

A close analysis of Table 2 indicates that the 36 profile HMMs allowed tracking new candidate ORFs/proteins. Interestingly, 34 out of the 46 ORFs/proteins identified by the different profile HMMs were identified by more than one profile HMM, while the remaining 12 were identified only once. The most frequently identified ORFs/proteins were: PVX_117880 (27 times), PVX_092975 (18 times) and PVX_002510 (17 times). On the other hand, PVX_080305, PVX_112665, PVX_092995, PVX_081810, PVX_101505, PVX_094425, PVX_086850, PVX_081845, PVX_097710, PVX_123550, PVX_109280, and PVX_118525 were identified only once. The profile HMMs labeled as “42, Pisces 90% identity”, “67, Cd-hit 30% identity”, “88, Cd-hit 30% identity” and “118, Pisces 30% identity” in Table 2 were those that identified the largest number of ORFs/proteins (12 in total).

Regarding the negative set, containing non-secreted reported ORFs/proteins used as a probe to validate the consistency of the results, only PVX_090110 was identified as a non-secreted protein out of the initial set of target proteins. This was the only case in which all profile HMMs identified the same protein.

Table S3 shows the protein domain families indexed in PFAM (version 25.0), which were reported by the hmmscan algorithm as being related to each built data set's protein sequences. Specifically, 310 domains were scanned in the PFAM database. The AMA-1 Apical membrane antigen 1, DUF605 Vta 1 like and MSP7_C MSP7-like protein C-terminal domains were the most retrieved domains (32 profile HMMs), while the HMM 129 (Cd-Hit 90%) profile scanned the highest number of domains (171 domains in total) (Table S3).

It should be stressed that the negative profile HMM scanned 164 domains that were not recognized by the positive profiles; 139 domains were thus recognized by the positive profiles but not by the negative ones. There were only 7 domains, which were recognized by both positive and negative profile HMMs (Table S3).

### Phase 3: Selection step

The 46 proteins identified in the target data set (45 positive and 1 negative) were compared against those reported in the literature and analyzed in terms of several structural features such as the presence of putative classical and non-classical signal sequences, the number of transmembrane helices, the presence of GPI-anchor sites, as well as the presence of protein domains relevant for invasion of erythrocytes and able to mediate cell adhesion (Table 3). The final categorization included 13 proteins which had already been classified as vaccine candidates according to previous studies; 9 of these have been tested for their antigenicity [21]–[25], two have been assayed for their protection-inducing ability [5], [21], [26], [27] and strong reticulocyte-binding ability has been described for the remaining two [28], [29]. Experimental evidence of protein expression has been published for 7 additional ORFs; however, no immunological or functional assays testing their potential role as vaccine candidates have been reported yet [5], [30]–[32]. Interestingly, 25 ORFs for which there is no additional experimental evidence, apart from their transcriptional profile, were also identified. This last group plus that containing the seven proteins for which immunological studies are lacking thus became interesting protein candidates to be further tested in vaccination assays (giving a total of 32 selected). The last protein out of the 46 classified was the negative one (Table 3).

**Table 3:** Computational protein selection results.

Protein ID	E. Ref	Category	NAME	ESLPred2	BaCelLo	SignalP	TM Pphob	SP Pphob	SecretomeP	TMHMM	GPI	DOM	MAAPs
PVX_123105	-	Selected	hypothetical protein, conserved	Ext	Sec								
PVX_101605	-	Selected	hypothetical protein	Ext	Sec		2		X	2	PFP		
PVX_123550	-	Selected	hypothetical protein, conserved	Ext	Sec				X				
PVX_118525	-	Selected	hypothetical protein, conserved	Ext	Sec	1–18	1	1–20		1			
PVX_081810	-	Selected	hypothetical protein, conserved	Ext	Cyt								
PVX_094425	-	Selected	hypothetical protein, conserved	Ext	Mit	1–27	11	1–27		9			
PVX_092425	-	Selected	hypothetical protein, conserved	Ext	Sec		1	1–23			HP		
PVX_081845	-	Selected	hypothetical protein	Ext	Sec		3		X	2			
PVX_080305	-	Selected	hypothetical protein, conserved	Ext	Sec	1–22		1–22					
PVX_101555	-	Selected	hypothetical protein	Ext	Sec	1–27	3	1–27		3			
PVX_002510	-	Selected	Nucleosomal binding protein 1, putative	Cyt	Sec		1			1			X
PVX_096990	-	Selected	Pv-fam-d protein	Ext	Sec	1–23	1	1–23		2			
PVX_117230	-	Selected	Ser/Thr protein phosphatase family protein	Ext	Sec		2	1–21					
PVX_101505	-	Selected	Pv-fam-d protein	Ext	Nuc		2			2			
PVX_092975	-	Selected	erythrocyte binding protein 1	Nuc	Sec	1–18	1	1–18		1		X	X
PVX_003825	-	Selected	serine-repeat antigen 4 (SERA)	Ext	Sec	1–26		1–24				X	
PVX_003805	-	Selected	serine-repeat antigen (SERA), putative	Ext	Sec	1–22		1–19				X	
PVX_003850	-	Selected	serine-repeat antigen 2 (SERA)	Ext	Sec	1–21						X	
PVX_097715	-	Selected	merozoite surface protein 3 (MSP3), putative	Ext	Sec	1–18		1–18			PFP		
PVX_097705	-	Selected	merozoite surface protein 3 alpha (MSP3a), putative	Ext	Sec	1–20		1–21					
PVX_097675	-	Selected	merozoite surface protein 3 gamma (MSP3g), putative	Ext	Sec	1–20		1–20					X
PVX_003830	-	Selected	serine-repeat antigen 5 (SERA)	Ext	Sec	1–24		1–22				X	
PVX_086850	-	Selected	variable surface protein Vir35, putative	Ext	Sec		3			3			
PVX_003815	-	Selected	serine-repeat antigen (SERA), truncated, putative	Ext	Cyt				X				
PVX_003905	-	Selected	transmission-blocking target antigen Pvs230, putative	Ext	Sec	1–29		1–22				X	
PVX_121885	5	Selected-PE	cytoadherence linked asexual protein, CLAG, putative	Ext	Sec	1–24	3	1–24		1		X	
PVX_084720	5	Selected-PE	hypothetical protein, conserved	Ext	Sec	1–22		1–22					X
PVX_092995	5	Selected-PE	tryptophan-rich antigen (Pv-fam-a)	Ext	Sec	1–23		1–23					
PVX_112665	5	Selected-PE	tryptophan-rich antigen (Pv-fam-a)	Ext	Sec	1–28		1–28					
PVX_085930	5,32	Selected-PE	rhoptry-associated protein 1, putative	Ext	Sec	1–22		1–22				X	
PVX_082695	31	Selected-PE	merozoite surface protein 7 (MSP7), putative	Ext	Sec	1–20		1–22					
PVX_117880	30	Selected-PE	hypothetical protein, conserved	Ext	Sec	1–17	3	1–17		1			
PVX_097670	22	VC-AA	merozoite surface protein 3 gamma (MSP3g), putative	Ext	Sec	1–20		1–20					X
PVX_121920	28	VC-FA	reticulocyte-binding protein 2 (RBP2), like	Ext	Sec	1–21	1	1–21					
PVX_097590	26	VC-PIA	rhoptry-associated protein 2, putative	Ext	Sec	1–21		1–21					
PVX_109280	24	VC-AA	tryptophan-rich antigen (Pv-fam-a)	Ext	Sec	1–25		1–22					
PVX_090210	10	VC-AA	hypothetical protein	Ext	Sec	1–20		1–20			PFP		
PVX_099980	5,10,27	VC-PIA	major blood-stage surface antigen Pv200	Ext	Sec	1–19	1	1–19		1	HP	X	X
PVX_097680	10	VC-AA	merozoite surface protein 3 beta (MSP3b)	Ext	Sec	1–19		1–19					X
PVX_097700	10	VC-AA	merozoite surface protein 3 (MSP3), putative	Ext	Cyt								X
PVX_097695	10	VC-AA	merozoite surface protein 3 alpha (MSP3a), putative	Ext	Sec	1–20		1–21					
PVX_097710	10	VC-AA	merozoite surface protein 3 (MSP3), putative	Ext	Cyt								X
PVX_003800	25	VC-AA	serine-repeat antigen (SERA)	Ext	Sec	1–25		1–20				X	
PVX_097720	23	VC-AA	merozoite surface protein 3 alpha (MSP3a)	Ext	Mit	1–23		1–23					X
PVX_098585	28,29	VC-FA	reticulocyte-binding protein 1 (RBP1), putative	Ext	Cyt	1–22	1	1–21					
PVX_090110	5	Negative-PE	hypothetical protein, conserved	Ext	Cyt		3						

Abbreviations: E. Ref. refers to the published reference where the relevant experimental data have been published for each protein. VC: Protein classified as Vaccine Candidate according to previous studies. PE: Experimental evidence of protein expression. PIA: Previous protection-induction assays. AA: Previous antigenicity assays. FA: Previous functional assays. ESLPred2 (Ext = extracellular, Cys = cytoplasm, Nuc = nuclear), BaCelLo (Sec = secretory pathway, Cyt = cytoplasm, Mit = mitochondrion, Nuc = nucleus), GPI (HP = highly probable, PFP = potential false positive), DOM = domains.

Importantly, 18 out of the 32 selected proteins were predicted as being secreted via the classical pathway to the extracellular medium and having a signal peptide, while three additional ones' secretion seems to occur via the non-classical pathway, according to at least four prediction tools. Predictors yielded contradictory results for the 11 remaining proteins.

## Discussion

Adjusting and modifying the algorithm parameters shifted the probability of the appearance of biological variables represented in the sequences, thereby broadening the identification of molecules likely to be far-relatives within the target protein data set. It should be noted that, although the data used for constructing the protein profile HMMs were similar (Table 1), the results obtained differed for all profiles so constructed (Tables 2 and 3).

Probabilistically, when a data set is analyzed by means of a redundancy adjustment algorithm, then it would be assumed that, depending on the extent of decreased identity percentage values, there should be more diversity among results. Different identity percentage parameters were thus explored, seeking to favor sequences which, while being different, did share some degree of similarity. The methodology followed in protein set definition and adjustment was thus designed considering that profiles adjusted to 30% identity would be more diverse in terms of the variables included in them. However, since the data sets for constructing the profile HMMs were so limited according to the selection criteria, it was difficult to establish such relationship.

As for the set of the 45 proteins identified, the presence of several of them was somehow expected considering that they belong to families that have already been characterized as good malaria antigen candidates; specifically, 6 proteins belonged to the serine repeat antigen family (SERA) which is considered a vaccine candidate antigen for *P. falciparum* malaria due to the immunogenicity conferred in rats by one of them when expressed as a recombinant protein [33]. It is worth noting that all 6 proteins identified as being SERA were *P. falciparum* SERA1, SERA2, SERA3, SERA4, SERA5, SERA6, SERA7 and SERA9 orthologs (Table S4). This group also included two ORFs identified as being rhoptry-associated proteins (RAP1 and RAP2). Some of the previously identified and characterized rhoptry proteins have been found to be actively involved in red blood cell invasion as they are able to bind to red blood cells or since monoclonal or polyclonal antibodies raised against them have inhibited *in vitro* invasion of target cells [32], [34]. An additional rhoptry protein (RON2) [30] expressed in the rhoptry necks was also identified. Previous studies have shown that the RON2 ortholog in *P. falciparum* is essential for parasite invasion due to its interaction with AMA-1 [35].

The profile HMMs also identified reticulocyte binding proteins (RBP1 and RBP2) which are directly associated with *P. vivax* merozoites' preference for invading human reticulocytes [28]; 9 MSP3 and 1 MSP7 (merozoite surface proteins 3 and 7, respectively) were similarly identified. MSPs are among the best candidate antigens for inclusion in an antimalarial vaccine, mainly because their surface localization facilitates their initial attachment to the red blood cell and involvement in subsequent invasion [36]. Moreover, this localization leaves them accessible to interact with host antibodies.

Regarding the protein identified as negative, it should be noted that it was only identified by the four probabilistic profiles constructed from the data set of 129 proteins, using the redundancy reduction algorithms at 30% and 90% identity (Table 2).

Of the 32 “selected” proteins (Table 3), 26 have orthologs in other species from the Plasmodium genus but the present analysis focused on 21 of them having orthologs in *P. falciparum*. The list of orthologous proteins can be found in Table S4.

Although tools predicting protein localization within a broader range of cellular organelles in eukaryotes are currently available, it has been particularly difficult to define which motifs or domains are exclusively associated with proteins located in apical organelles [37]. The only consensus regarding signals targeting proteins to cell surface or to apical compartments so far consists of the presence of a classical signal sequence or, eventually, the presence of some secondary targeting signals [38], [39]. This led us to perform an additional analysis of the set of 45 identified proteins to define priorities in identifying new antigens which are potentially involved in *P. vivax* invasion of reticulocytes. It was thus ascertained whether proteins had a classical signal peptide sequence, as well as the presence and number of transmembrane regions, GPI-anchor sites and domains known to be involved in host-cell invasion, and their ability to mediate cell adhesion.

It has been observed that parasite antigens involved in host-cell invasion that are targeted to membrane or apical organelles are stabilized within such organelles via transmembrane helices or, if such regions are lacking, then by their non-covalent association with other anchored-proteins [39].

It is clear that an important number of *P. falciparum* cell surface-associated proteins are attached to the plasma membrane via GPI anchors [40]. Is also well known that some of these proteins play an active role in host-cell invasion through their incorporation into lipid rafts [41], also being recognized by neutralizing antibodies induced during natural malarial infection in people living in endemic areas [42]. Based on these data, various GPI-anchored proteins, such as MSP1 and MSP2, have been or are currently being assessed as candidates for a vaccine against this parasite species [43]. Given the importance of proteins containing GPI-anchor sites, understood as a post-translational modification, a GPI-anchored prediction was thus run on the set of 45 proteins identified using the FragAnchor tool [44]. Such analysis predicted a highly probable (HP) GPI-anchor attachment site in 2 of these proteins and 3 had a potentially false positive GPI-anchor site (Table 3).

Despite the difficulty in determining consensus domains and motifs of apical organelle proteins in members of the phylum Apicomplexa, the 45 proteins selected in the computational protein identification were screened against the CODD database [15]. Among the domains used as search query, only those potentially involved in host-cell invasion by mediating adhesion to receptors on the target cell surface or actively involved in shedding-off other parasite proteins were included. This search for *P. vivax* proteins containing one or more of these domains was carried out using PlasmoDB v6.1 based on a list of accession codes for the domains reported in PFAM [45] and InterPro [46] (Table 3).

Another alternative was to check which selected proteins were predicted to act as adhesins. The MAAP [47] tool was thus used which was designed on the sequence composition of a group of proteins from the genus Plasmodium, experimental evidence having shown them to be directly involved in adhesion to host cells (Table 3).

It should be noted that even though the performance of the approach chosen here depended on the current status of parasite genome annotation, the exploratory methodology focused on identifying true positive values and therefore sought to optimize search precision rate. The results are thus not to be measured by the number of proteins so identified but rather by the method's reliability in confirming that such identified proteins are actually true positives. In other words, the fraction of true vaccine candidates among those that the algorithm identified as belonging to the candidates subset had to be close to one.

The information made available through the publication of the genome sequences of several parasites and their hosts, as well as of malaria vector mosquitoes, together with analyses focused on the detection and quantification of parasite gene transcription and protein expression, is now allowing novel strategies to be postulated and better approaches to be adopted for designing more effective vaccines, drugs, diagnostic methods and treatments [48]. This study constitutes an exploratory and rational approach toward the identification of *P. vivax* proteins playing a potential role in invasion of human reticulocytes based on the search for far-homologues by applying algorithmic techniques for adjusting sequences in regards to redundancy, construct profiles based on HMMs and analyzing protein features such as amino acid composition and secretion pathways.

## Materials and Methods

The bioinformatics approach proposed in this paper explored a methodology for identifying *P. vivax* proteins that could be involved in parasite invasion of reticulocytes, therefore making them interesting vaccine candidates. The methodology was based primarily on the use of techniques for reducing sequence redundancy and constructing HMM-based profiles. The main purpose of the methodology was the construction of various probabilistic profiles to search for biologically-related proteins in the *P. vivax* intra-erythrocyte transcriptome. The probabilistic profiles were built using different data sets with sequences from the Plasmodium genus (9 sets in total (Table S1), each with 4 profiles, for a total of 36 profile HMMs) generated by varying protein feature parameters, and which were adjusted using 2 different methods to reduce redundancies. Proteins identified as possible candidates were further analyzed according to their sequence and additional features to select the most likely vaccine candidates (Figure 1).

The approach followed in this study can be divided into 3 main phases (Figure 1). The objective of the first two phases was to identify proteins potentially involved in *P. vivax* invasion of reticulocytes. These stages involved generating suitable data sets (phase 1a), eliminating protein sequence redundancy (phase 1a), generating protein alignments and obtaining probabilistic profiles based on HMMs (phase 1a), generating the 582 *P. vivax* target data set (phase 1b) (Table S2) and scanning the target data set with the profile HMMs (phase 2). The protein set obtained after these stages included previously reported proteins that had been described as participating in *P. vivax* invasion but, more interestingly, it also contained proteins that have not been previously implicated in cell invasion.

The third phase, denoted as the selection step, consisted of debugging the data set obtained in the previous stages to select the most likely vaccine candidates. This included reviewing scientific literature in the search for previous experimental evidence regarding vaccine candidates or expressed proteins. In addition, the identified proteins were analyzed using different computational classifiers to detect cellular secretion, signal peptides, transmembrane regions, GPI-anchor regions, invasion-related domains and adhesion-like proteins. The orthologs for all the proteins identified are reported in Table S4. A detailed description of each step followed in these 3 phases is given below.

### Phase 1: Constructing protein profile HMMs and the target data set

#### Constructing the protein sequences

Positive and negative sets of proteins were created based on a literature review and search in biological databases (Table 1). In the case of the positive data set, 8 subsets of non-exclusive proteins were formed to model the possible values for a protein's attributes and the organism to which it belonged. Specifically, the attribute “Protein existence” from the SwissProt database was modeled by generating data sets representing each possible value for that attribute (“predicted” and “putative or hypothetical”). Similarly, the organism to which the protein belonged was modeled by including the four species of *Plasmodium* considered here. The negative set consisted of proteins having an annotation of being localized in cytoplasm, nucleus, mitochondria, or retained in the endoplasmic reticulum, whereas the positive set clustered proteins targeted at *P. falciparum*, *P. vivax*, *P. berghei* and *P. yoelii* cell surface, rhoptries or micronemes.

Table S1 shows the 9 different data sets created for the exploration. It should be noted that there was no exclusive set of proteins for each data set, but rather proteins were shared between more than one set.

The set construction generated sets having divergent features (Table 1). Specifically, the number of sequences ranged between 133 sequences (Set 2) and 20 sequences (Set 9). Some of the sets resulted from combining searches in databases and literature reviews (Sets 3 and 5 to 9) while other sets were built strictly on query results from databases (Sets 1, 2 and 4). The parameter of “existence” diverged between the sets, allowing for some of them to contain proteins for all possible values (Sets 2, 3 and 7) whereas others were restricted to proteins combining two of these annotations (Sets of 4, 5 and 8) and others were restricted to proteins with one particular type of annotation (Set 6 and 9). Regarding distribution in the organism of origin, some sets were unbalanced in favour of *P. falciparum* (Sets 1 to 7) while others tended to cluster proteins from *P. vivax* (Sets 8 and 9).

It is worth noting special features for certain data sets. For example, Set 7 clustered 67 *P. falciparum* proteins reported as being involved in invasion and with annotations of function, subcellular localization and time during the cycle in which they are transcribed [49]. Similarly, all proteins present in the data sets were compared with the target set and were extracted before constructing the profiles. This step was carried out to avoid biasing profile construction by favoring sequences repeated between a specific profile and the target set.

An essential premise was that the divergence among the data sets reported in Table 1 allowed generating different probabilistic profiles, therefore leading to different search approaches within the target data set. The negative set was used to validate the robustness of the results obtained in the exploration of the target data set.

#### Reducing redundancy

The task was performed by applying the Cd-hit [19] and PISCES [20] algorithms to each of the 9 protein profile data sets (defined in the previous subsection), according to 2 identity parameters (30% and 90% identity). This reduction of redundancy resulted in a total of 36 subsets.

#### Constructing protein profile HMMs data sets

This stage consisted of obtaining protein alignments. Sequence alignments for the 36 subsets (defined in the previous subsection) were obtained using the ClustalW2 suite [50].

A profile HMMs comparison between the different data sets and the PFAM 25.0 database was also performed. Specifically, the ‘hmmscan’ algorithm in the HMMER v3.0 suite [18] was the choice selected for a protein sequence vs profile-HMM database query. Table S3 shows the domains reported as being related to data set protein sequences. The profile HMMs are publicly available in http://www.biolisi.unal.edu.co/publications/supplementary-files/p_vivax/.

#### Target data set

This set was constructed on *P. vivax* gene mRNA abundance levels during each time interval during the intra-erythrocyte cycle, expressed in terms of the degree of RNA hybridization in the microarray compared to hybridization in the RNA reference pool assembled from the RNA samples from all time points for the 3 studied isolates [4]. Of the total of 5,335 genes analyzed in the 3 clinical isolates, only those genes showing a Cy5/Cy3 ratio higher than 1.5 in the TP7–9 time interval (corresponding to 31–48 hours of intra-erythrocyte cycle), but not in any of the previous time intervals (TP1–6), were selected. This selection considered genes transcribed in at least one *P. vivax* isolate to discard genes that did not have a single maximum transcription peak at the end of the intra-erythrocyte cycle.

It should be noted that genes having larger transcription peaks at any previous time (0–30 hours) were not taken into account. The transcription criteria “at the end of the intra-erythrocyte cycle” was chosen due to previous evidence showing that most *P. falciparum* genes encoding proteins having important roles in erythrocyte invasion have a peak transcription during this lapse of time, which agrees with merozoite maturation and the development of apical organelles (rhoptries, micronemes and dense granules) [51]. This resulted in a target data set of 582 transcribed genes (Table S2).

All amino acid sequences used in this study, either identified previously or as a result of *P. falciparum* and *P. vivax* genome annotation were downloaded from the PlasmoDB database [52], with their corresponding accession codes.

### Phase 2: Protein profiles against target data set and protein identification

The probabilistic profiles were obtained and used as query for searching in the target protein data set using HMMER v 3.0 software [18]. The results for this phase are reported in Table 2.

### Phase 3: Protein selection

The protein identification described in the previous section defined a set of 46 candidates (Table 3). This set of proteins was further characterized according to structural features regarding secretion and the transcriptional profile reported in PlasmoDB. These results were then contrasted with previous literature reports to classify proteins already being considered as vaccine candidates and those that were not. As a result of this analysis, the identified proteins were grouped into two categories: “vaccine candidates” and “selected”. The respective sub-categories can be found in Table 3.

The *P. vivax* gene homologues located in syntenic regions were identified in the annotated *P. falciparum* genome by screening the PlasmoDB [52] and the OrthoMCL–DB [48] databases (Table S4). The subcellular localization of the pool of proteins selected based on the abovementioned criteria was also predicted using tools based on Support Vector Machine (SVM) systems and/or homology-based analyses. The ESLpred2 tool [53] hosted at http://www.imtech.res.in/raghava/eslpred2/and BaCelLo [54] available at http://gpcr.biocomp.unibo.it/bacello/were used, selecting the animal kingdom-specific predictor in both cases.

The next step was to screen proteins for the presence of a signal peptide using various tools for predicting the secretion pathway in eukaryotic proteins. SignalP 3.0 [55] http://www.cbs.dtu.dk/services/SignalP/was used for classical secretion as it combines neural networks (NN) and hidden Markov models (HMM), as well as PolyPhobius [56] http://phobius.sbc.su.se/poly.html, a tool that allows determining transmembrane topology performing an HMM algorithm using homology information. Secretion via non-classical pathways was predicted using SecretomeP 2.0 [57] available at http://www.cbs.dtu.dk/services/SecretomeP/.

Transmembrane helices and GPI anchors were also reported as part of the analysis. PolyPhobius and TMHMM v 2.0 [58] http://www.cbs.dtu.dk/services/TMHMM/were used for predicting the presence and number of transmembrane regions in the pool of chosen peptide sequences. The latter tool is based on the use of an HMM; however, unlike PolyPhobius, TMHMM sometimes incorrectly classifies the signal peptide as a transmembrane region. Those regions predicted as transmembrane helices but showing at least 50% similarity with one or both of the signal peptide predictions mentioned above were thus discarded. All proteins were analyzed for the presence of GPI-anchor sites using FragAnchor (http://navet.ics.hawaii.edu/~fraganchor/NNHMM/NNHMM.html) [44] which is based on the tandem use of NN and HMM predictors.

*P. vivax* proteins having a potential adhesive function, being understood as a protein's ability to adhere to another protein or molecule on red blood cell surface, were identified using the Malarial Adhesins and Adhesin–like proteins (MAAP) predictor [59] available at http://maap.igib.res.in/, which is a non-homology-based approach that implements the compositional characteristics of amino acid dipeptides and multiplet frequencies with an SVM-based classification system.

## Supporting Information

Table S1 **Profile data sets.**
(XLS)

Table S2 **Target data set.**
(XLS)

Table S3 **Pfam domains related to the data sets constructed.**
(XLS)

Table S4 **Ortologs of the identified proteins found in other Plasmodium species.**
(XLS)
